# Improvement of functional characteristics of *Hypophthalmichthys molitrix* protein by modification with chitosan oligosaccharide

**DOI:** 10.3389/fnut.2023.1140191

**Published:** 2023-05-26

**Authors:** Haihua Cong, Qiming Wu, Zhuoran Zhang, Juntao Kan

**Affiliations:** ^1^School of Food and Drug, Shanghai Zhongqiao University of Vocational Technology, Shanghai, China; ^2^Nutrilite Health Institute, Shanghai, China

**Keywords:** chitosan oligosaccharide, myofibrillar protein, myosin, functional, silver carp

## Abstract

In the food processing field, it is very often that fish proteins are denatured affecting the nutritional value of the product which is vital to be solved. By using appropriate sugar donors for glycosylation with protein, improving the stability and emulsification properties of fish proteins can be achieved. This research looks into the impacts of enzymatic chitosan oligosaccharide (CO) at various concentration (0.15%, 0.30%, 0.45%, 0.60%, w/v) upon the molecular makeup and function of silver carp myofibrillar protein (MP) in an attempt to comprehend the impact of electrostatic binding among MP as well as CO on protein conformation. Analysis was done on the impact of various CO concentrations upon MP’s secondary structure, conformational changes, and functional characteristics. Twelve sodium dodecyl sulfate polyacrylamide gel electrophoresis (SDS-PAGE) assays were implemented to monitor MP; Fourier transform infrared spectroscopy, endogenous fluorescence spectroscopy, and UV absorption spectra were carried out to investigate the influence of CO on MP; Particle size distribution, emulsifying activity index (EAI), solubility, turbidity, sulfhydryl content, carbonyl content, foaming capacity, surface hydrophobicity, emulsifying stability index (ESI), and foam persistence were all investigated. In addition, we used dynamic light scattering, scanning electron microscope, and atomic force microscope to analyze myosin (MO) and 0.60% CO-MO complex. The results demonstrated that CO and MP form complexes through hydrogen bonding and electrostatic interactions. CO modification not only delayed the oxidation of MP but also promoted MP to show better solubility, foaming, and foaming stability. In addition, CO modified myosin particle size decreased, reducing myosin’s roughness and making myosin’s structure more compact. To sum up, molecular interaction could change functional characteristics, and products with special properties could be developed after modification with chitosan oligosaccharide.

## 1. Introduction

Fish protein is one of the important protein sources in the diet and is abundant in amino acids. The principle element from fish protein contain myofibrillar proteins (MP), which have great functional qualities like that of the ability to create gels and retain water (1). However, throughout preparation, treatment, and preservation, certain chemical qualities and low thermal stability of fish myofibrillar proteins result in decreased MP solubility and emulsifying properties. To enhance fish protein’s functional characteristics and yield better food, it is therefore crucial to prevent or minimize denaturation during both food preservation and preparation (2). The most recent techniques for strengthening the functional characteristics of proteins include: enzymatic modification; chemical techniques like the use of sodium alginate (SA) and L-histidine, acid, small-molecule compounds or proteases; and physical techniques like high-intensity ultrasound (HIU), high pressure processing (HPP), high pressure homogenization (HPH), superfine grinding (SG), washing, and dilution (1).

It is feasible to add new activities to fish proteins and enhance their essential features through glycosylation (3). The emulsifying characteristics and dietary proteins’ thermal stableness could be gotten better through glycosylation as well as alginic acid oligosaccharide or glucan, for example, which also increased the solubility for carp MP when joined in ribose (4). Glycosylation is its bond attachment of carbohydrates to amino groups that are accessible in proteins, and it is used in the Amadori rearrangement step of the Maillard process to produce new glycoproteins. In contrast to different types of alteration, the glycosylation reaction takes place in fairly secure conditions. Because glycosylation process does not require extra compounds, it is preferable to use it in manufacturing of food and beverages (5).

The selection of the proper glycosyl donor to engage with the protein is a crucial stage in the glycosylation process. Studies have shown that the solubility of MP is significantly improved by glycosylation, such as ribose or glucose. The protein’s thermal stability and emulsifying properties were greatly improved when dextran was used as the sugar donor. Choosing small molecule sugar donors with the appropriate molecular mass, polymerization level, and structural characteristics is considered important (4). Chitosan oligosaccharide (CO) is manufactured by depolymerization of chitosan (e.g., acid hydrolysis, redox hydrolysis, enzymolysis, or high-energy cleavage). CO promoted the water solubility of protein and decreased the solution viscosity of protein. CO modified by the Maillard reaction has a variety of biological and pharmaceutical applications. CO and β-lactoglobulin can effectively enhance the antioxidant activity of the complex. Whey protein isolate’s solubility, thermal conductivity, and emulsification qualities were all increased by CO, and tropomyosin’s allergenicity was diminished (6–8).

Currently, many reports have focused on the fundamental research and applications of using the electrostatic interactions among polysaccharides and proteins from numerous sources to enable the building into complex condensates at different scales (9). However, the strength of the interface performance of protein polysaccharide complex condensate depends not only on the scale effect and physicochemical properties of the complex but also on the degree of conformational transformation of protein in the process of electrostatic binding (4, 10–12). Consequently, the crucial thing turns out to be to look into the effect of electrostatic binding with polysaccharides upon that configuration of protein molecules as well as to further understand the properties of interfaces and the process of complex condensates.

**Figure 1:**
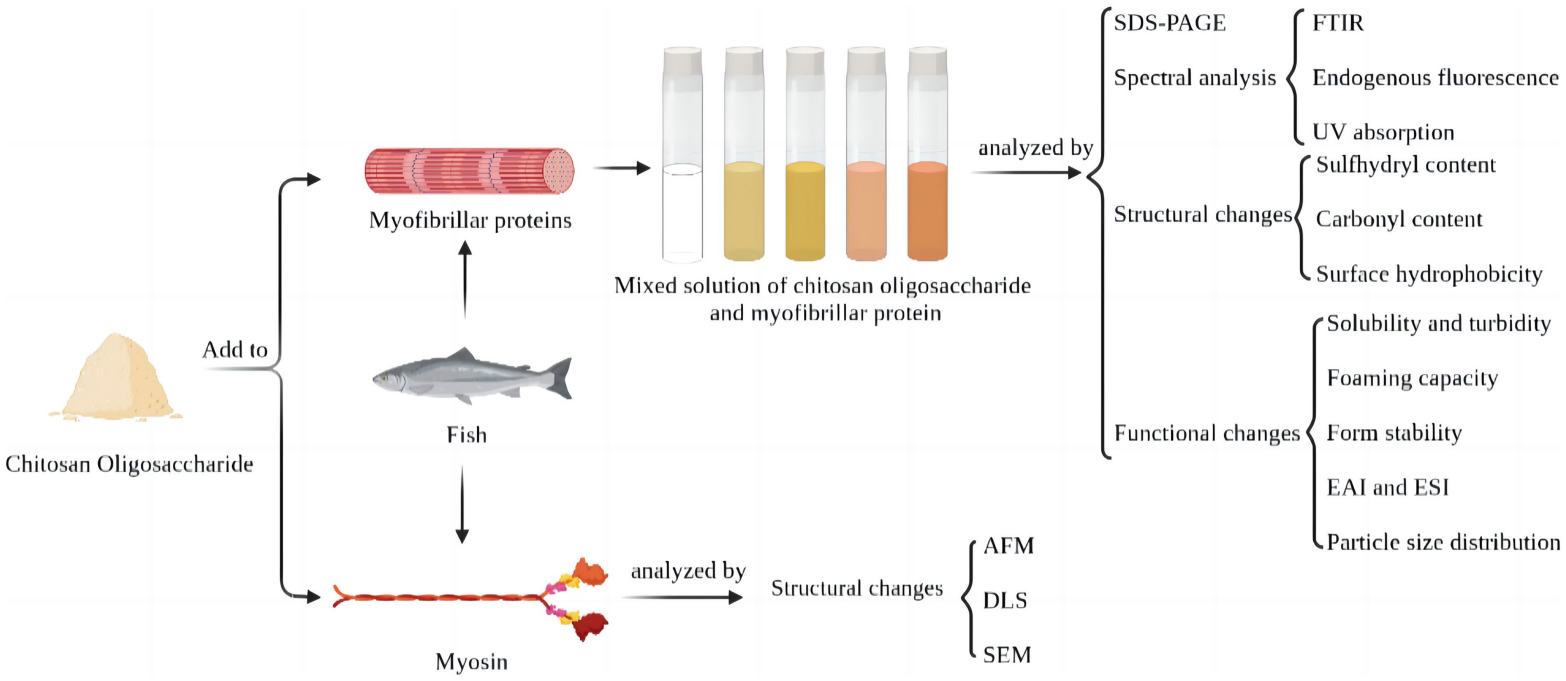
Schematic illustration of improvement of functional characteristics of silver carp protein by modification with chitosan oligosaccharide.

Whereas, the effect of electrostatic binding between MP and CO on protein conformation has not yet been revealed, which is vital in further to understand the mechanism of the MP/CO complex stabilizing the lotion and proposing relevant regulatory strategies (13, 14). Throughout this research, UV scanning spectroscopy, internal fluorescence spectroscopy, as well as Fourier transform infrared spectroscopy were performed to look into potential effects of oligosaccharides on myofibrillar proteins. Moreover, 12 sodium dodecyl sulfate polyacrylamide gel electrophoresis (SDS-PAGE), sulfhydryl content, carbonyl content, surface hydrophobicity, solubility, turbidity, foaming capacity, foam stability, emulsifying activity index (EAI), emulsion stability index (ESI) and arrangement of particle sizes had been undertaken, and such impact of distinct densities of chitosan oligosaccharide on the secondary structure, conformational changes, and function of myofibrillar protein were also analyzed. In addition, we also analyze the effect of 0.60% chitosan oligosaccharide on myosin by using dynamic light scattering, atomic force microscope, and scanning electron microscopy (Figure 1). This study can further analyze the interface mechanism of CO-MP complexes, and offer fresh perspectives for enhancing proteins’ functional characteristics after electrostatic binding to functional oligosaccharides with proteins in the process of glycosylation, which is convenient for future researchers to conduct further exploration in this field.

## 2. Materials and methods

### 2.1. Materials and reagents

Enzymatic chitosan oligosaccharide was given access through the Institute of process engineering, Chinese Academy of Sciences; the fresh silver carp was purchased from the Dalian food and cooked food trading center and transported to the laboratory within 40 min. After slaughter, the head and viscera were removed and washed for standby.

Sodium dodecyl sulfate (SDS) was provided by Tianjin Damao Chemical Reagent Factory (Tianjin, China); DTNB, DNPH, TCA and ANS were provided by Shanghai Macklin Biochemical Co., Ltd. (Shanghai, China); EDTA was provided by Tianjin Zhiyuan Chemical Reagent Co., Ltd. (Tianjin, China); β-mercaptoethanol was provided by Shanghai Yaji Biotechnology Co., Ltd. (Shanghai, China). Potassium bromid was spectrally pure, all other chemicals were analytical grade and provided by Sigma-Aldrich (St. Louis, MO, USA) Sigma-Aldrich (St. Louis, MO, USA) or Sinopharm Chemical Reagent Co., Ltd. (Shanghai, China).

### 2.2. Instruments and equipment

DS-1 high speed tissue crusher, Shanghai specimen model factory; HR/T 20 mm vertical high-speed freezing centrifuge, Hunan Hexi Instrument Equipment Co., Ltd.; Synergy h1 / h1m microplate reader, American Berton Instrument Co., Ltd.; F-2700 fluorescence spectrophotometer, Hitachi, Japan; Nexus670 infrared spectrometer, PerkinElmer, USA; UV-9000 double beam UV–Vis spectrophotometer, Shanghai yuanxi Instrument Co., Ltd.; Scientz-30D vacuum freeze dryer, Ningbo Scientz Biotechnology Co., Ltd., China; HH-6 Digital display thermostatic water bath, Guohua Electric Appliances Co., Ltd., China; FJ300-SH Digital display high-speed dispersion homogenizer, Shanghai Biaoben Mould Factory, China; Tescan Vega3 Scanning Electron Microscope, Hitachi Ltd., Japan; Mastersizer 2000 Particle size analyzer, Malvern Instruments UK Co., Ltd., UK; Atomic force microscopy, Bruker Co., Ltd., German.

### 2.3. Methods

#### 2.3.1. Extraction of myofibrillar protein

Remove silver carp’s back tenderloin by hand, cut it into minced meat, add 5 times the volume of 10 mmol L^−1^ Tris–HCl (pH 7.2) buffer, homogenize it at 4,000 r min^−1^ for 2 min, after that centrifuge it at 5,000 r min^−1^ at 4°C until 15 min; Precipitate in 5 times volume of 10 mmol L^−1^ Tris–HCl (containing 0.6 mol L^−1^ NaCl, pH 7.2) buffer, homogenize at 4,000 r min^−1^ for 30 s, centrifuge at 4,500 r min^−1^ at 4°C for 20 min, after that take supernatant, which is myofibrillar protein. A biuret reagent has been utilized to measure the concentration (15).

#### 2.3.2. Preparation of composite

The approach was founded on Yang et al. (16). Dilute the extracted myofibrillar protein with 10 mmol L^−1^ Tris–HCl (containing 0.6 mol L^−1^ NaCl, pH 7.2) buffer to 10 mg mL^−1^, and put it into a centrifuge tube for standby. Add CO of different concentrations (0.00%, w/v, 0.15%, w/v, 0.30%, w/v, 0.45%, w/v, 0.60%, w/v), stand at 4°C, and measure after CO is fully swollen.

#### 2.3.3. Sodium dodecyl sulfate polyacrylamide gel electrophoresis (SDS-PAGE)

The crosslinking of CO-MP was judged using a transitory buffered system SDS-PAGE that comprised 5% stacking as well as 12% detaching gels (17). The loading capacity of each lane is 10 μL. The gel was stained with 0.1% (w/v). Gels were stained for 1 h with 0.1% (w/v) Coomassie Brilliant Blue R-250 in an 80% methanol and 20% acetic acid solution, and then destained for 2 h in a solution of 50% methanol and 10% acetic acid. By contrasting the band with the standard protein solution, the relative molecular weight standard of the band was identified.

#### 2.3.4. Fourier transform infrared spectroscopy

With a few change, the technique was altered according to Xu et al. (17) and Hu et al. (18). After freeze-drying, the dried sample powder was fully mixed with anhydrous potassium bromide in 1:50, pressurized for 2 min under the pressure of about 18 MPa, and the scanning range was 450 ~ 4,000 cm^−1^. Based on second derivative spectrum, gauss peak fitting is adopted, the integral area is used to estimate the protein secondary structure %. 1,651 ~ 1,600 cm^−1^ is considered as α-helix structure band, 1,600 ~ 1,639 cm^−1^ is regarded as β-sheet band, 1,661 ~ 1,700 cm^−1^ is considered as β-turn structure band, 1,640 ~ 1,650 cm^−1^ is considered as random coil band (12).

#### 2.3.5. Endogenous fluorescence spectrum

A fluorescence spectrophotometer was employed in order to analyze the solution’s fluorescence spectrum. Proteins’ fluorescence spectra of the were then examined at emission wavelengths of 300 to 450 nm and excitation wavelengths of 280 nm for different C3G concentration gradients. Measure the fluorescence intensity (19). Using stern Volmer equation:


$$\frac{F_{0}}{F}=1+K_{SV}\left[Q\right]$$

where: *F*_0_ and *F* are the highest fluorescence intensity of oligosaccharide free and oligosaccharide protein solution respectively; [Q] is the concentration of oligosaccharide (mg mL^−1^); *K*_SV_ is the quenching constant (20).

#### 2.3.6. UV absorption spectrum

Dilute the myofibrillar protein sample to 1 mg mL^−1^, take Tris–HCl (containing 0.6 mol L^−1^ NaCl, pH 7.2) buffer as blank, and obtain the ultraviolet absorption spectrum in the range of 230 ~ 600 nm. Use Originpro 2017 software to obtain the second derivative spectrum of UV absorption spectrum data (20).

#### 2.3.7. Active sulfhydryl

The method of active sulfhydryl content was determined according to Yildiz et al. (21) and slightly modified. Dilute the sample protein concentration to 4 mg mL^−1^: 0.5 mL of the sample and 4.5 mL of the reaction buffer (0.2 mol L^−1^ Tris–HCl, 10 mmol L^−1^ EDTA, pH 6.8) was mixed, add 0.5 mL of DTNB (10 mmol L^−1^) solution, react at 4°C for 1 h, and then measure the absorbance at 412 nm.


$$\mathrm{Sulfhydryl concentration}\;\left(\mathrm{mol}\;10^{-5}g\right)=\frac{\left(A-A_{0}\right)\times D\times 10^{5}}{13600\times C}$$

where: *A*: sample absorbance; *A*_0_: absorbance of blank sample; *D*: Protein dilution ratio: Molar extinction coefficient 13,600 (mol L^−1^)^- 1^ cm^−1^; *C*: Protein concentration.

#### 2.3.8. Carbonyl content

The method is slightly modified according to Bin Zhang et al. (22) research. The prepared myofibrillar protein sample was diluted to 2.0–2.5 mg mL^−1^, 0.5 mL of the sample was mixed with 1 mL of 10 mmol L^−1^ DNPH (2 mol L^−1^ HCl), and then reacted in darkness at room temperature for 1 h with vortexing every 10 min. Blank was prepared by adding an equal volume of 2 mol L^−1^ HCl as the replacement of DNPH solution. The mixture was precipitated with 20% (w/v; final concentration) TCA solution and centrifuged at 2,000 × g for 10 min at 4°C. The precipitate obtained by centrifugation (5,000 r·min^−1^, 4°C for 10 min) was washed thrice with 1 mL of ethanol: ethyl acetate (1:1, v/v). The ethanol/ethyl acetate extract was nearly discolored on the third wash, which indicated excess dinitrophenol was removed. The protein precipitate was then dissolved in 4 mL of 6 mol L^−1^ guanidine hydrochloride (dissolved in 20 mmol L^−1^ phosphate buffer) and incubated at 37°C for 20 min in a water bath. The supernatant was taken to measure the absorbance of the sample at 370 nm after centrifugation (10,000 r min^−1^, 5 min). The calculation formula of the carbonyl group is:


$$\mathrm{Carbonyl content}\;\left(\mathrm{mol}\;{\mathrm{mg}}^{-1}\right)=\frac{\left(A-A_{0}\right)\times D}{\varepsilon \times C}\times 10^{6}$$

where: *A*: absorbance of DNPH added; *A*_0_: absorbance of blank (DNPH not added); *D*: Protein dilution ratio: Molar extinction coefficient 22,000 (mol L^−1^)^−1^ cm^−1^; *C*: Protein concentration.

#### 2.3.9. Surface hydrophobicity

The surface hydrophobicity of the sample was determined referring to Li et al. (23) with slight modification. The samples were adjusted to different concentrations (0.1–0.5 mg mL^−1^) with 10 mmol L^−1^ Tris–HCl (0.6 mol L^−1^ NaCl, pH 7.2), and 2 mL of the adjusted samples were mixed with 10 μL ANS (8-anilino) -1-Naphthalenesulfonic acid (10 mM, pH 7.5) solution in a chromatographic cabinet. After incubating in darkness at 4°C for 20 min, the fluorescence value was taken (SynergyH1/H1M microplate reader), using an excitation wavelength at 375 nm, emission wavelength at 485 nm, and a gain of 50. Fluorescence intensity was plotted against protein concentration, and the slope of this line was designated as protein surface hydrophobicity (*S*_0_).

#### 2.3.10. Solubility

The MP solution was diluted with buffer to 5 mg mL^−1^, left standing at 4°C for 1 h, and centrifuged (5,500 r min^−1^, 4°C, 15 min). The protein concentration in the supernatant was measured with a biuret using bovine serum albumin as a standard protein (24).

#### 2.3.11. Turbidity

Samples were diluted to 1 mg mL^−1^ with 10 mmol L^−1^ Tris–HCl (containing 0.6 mol L^−1^ NaCl, pH 7.2) buffer. The turbidity of diluted samples was measured at a wavelength of 340 nm using the microplate reader (25).

#### 2.3.12. Foaming capacity and foam stability

The foaming capacity and stability were identified by referring to Ping-Ping et al. (9). Phosphate buffer (0.1 M, pH 7.4) was used to prepare sample solution. An FJ-180 high-speed homogenizer is used to homogenize (10,000 × g) for 5 min at room temperature, then the foaming capacity was expressed in terms of volume change rate. Foaming stability was expressed as the rate of change in foam volume after 20 min.

#### 2.3.13. Emulsifying activity index (EAI) and emulsion stability index (ESI)

The emulsifying activity and emulsifying stability were determined as reported by Yildiz et al. (21). Soy oil (1 mL) and 3 mL of CO-MP composite sample were homogenized (10,000 r min^−1^) to prepare oil-in-water (O-W) emulsions for 5 min and sonicated immediately for 5 min. The test oil concentration is 0.25% (w/w). Ultrasound is used to generate complex emulsions. After the emulsion was formed, the absorbance was measured at 500 nm at 0 min (*A*_0_) and 10 min (*A*_10_), respectively. The calculation formulas of emulsifying activity index (EAI) and emulsifying stability index (ESI) are as follows:


$$\mathrm{EAI}\left(m^{2}\;g^{-1}\right)=\frac{2\times T\times A_{0}\times D}{C\times \varphi \times L\times 10000}$$

$$\mathrm{ESI}\;\left(\min\right)=A0\times 10/\left(A0-A10\right)$$

where: *T*: 2.303; *D*: Dilution factor; *C*: Weight of protein per unit volume (mg mL^−1^); 50: Optical path width (0.01 M); *φ*: Oil volume fraction (0.25); Where *A*_0_ and *A*_10_ were the absorbance measured at 0 and 10 min, respectively.

#### 2.3.14. Particle size distribution

By using the laser light scattering method and Mastersizer 2000 instrument (Malvern Instruments Ltd., Worcestershire, UK), the volume-weighted mean particle diameter (*d*_4,3_) and surface-weighted mean particle diameter (*d*_3,2_) of the emulsions were measured. After storing the samples at room temperature (approximately 22°C) for 10 days (26). Emulsions containing different SE concentrations were diluted 100-fold with 0.01 M phosphate buffer (pH 7.0) to avoid multiple scattering effects. The *d*_4,3_ and *d*_3,2_ were calculated using the following equations:


$$d_{4,3}=\left(\Sigma n_{i}\;d_{i}{}^{4}/\Sigma n_{i}\;d_{i}{}^{3}\right)$$

$$d_{3,2}=\left(\Sigma n_{i}\;d_{i}{}^{3}/\Sigma n_{i}\;d_{i}{}^{2}\right)$$

where *d*_i_ is the droplet diameter and n_i_ is the number of droplets of diameter d_i_. The refractive indexes of the continuous and dispersed phases were set at 1.33 and 1.48, respectively.

#### 2.3.15. Myosin extraction

The method of myosin extraction process using these two references Cao et al. (27) and (28). The concentrations of myosin were diluted to 10.0 mg/mL using 0.5 mol/l KCl (20 mmol/l Tris–HCl, pH 7.5) buffer for storage and use, all analytical representations must be tested within 3 days.

#### 2.3.16. Atomic force microscope

The concentration of the myosin sample was adjusted to 1 mg mL^−1^, and an appropriate solution was applied in drops on the surface of the freshly peeled mica sheet and dried naturally at room temperature to form a myosin adsorption layer (29). Atomic force microscopy measurements were performed using the TAP 150 probe model (MPP-1210010) and the molecular force operating mode. The thickness of the microcantilever is *T* = 1.5–2.5 μm, *L* = 115–135 μm, *W* = 25–35 μm; the resonance frequency f0 is 150–200 kHz; the elastic coefficient *k* is 5 N m^−1^, and the resolution is 256*256.

#### 2.3.17. Dynamic light scattering

The particle size distribution of the protein was determined by dynamic light scattering using a ZS90 equipped with a 4 mw He-Ne ion laser (*l* = 633 nm) (29). The 1 mg mL^−1^ protein solution was placed in a 1 cm diameter quartz test tube at 25 ± 0.1°C with a detection angle of 90°.

#### 2.3.18. Scanning electron microscopy

A Tescan Vega3 SEM was used to observe the surface morphology of complex powder (200× magnification). Complex powder was evenly mounted on an aluminum stub, coated with a thin layer of gold, and observed using a cabin voltage of 20.0 kV (30).

### 2.4. Statistical analysis

All the samples were measured in triplicate and results were expressed as mean ± standard deviation (SD). Differences between mean values were conducted using the one-way analysis of variance (ANOVA) by SPSS 17.0 software. Significant differences were determined at *p* < 0.05.

## 3. Results and analysis

### 3.1. SDS-PAGE

**Figure 2:**
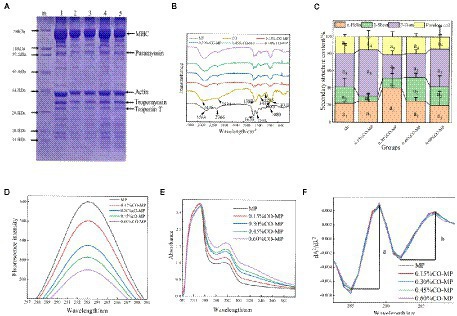
**(A)** SDS-PAGE patterns of a mixture of chitosan oligosaccharide (CO) and myofibrillar protein (MP) (Note: m is Markers; 1 is MP, 2 is 0.15% CO-MP, 3 is 0.30% CO-MP, 4 is 0.45% CO-MP, and 5 is 0.60% CO-MP) **(B)** Effect of CO on the Fourier transform infrared spectroscopy of MP **(C)** Effect of CO on the secondary structure content of MP **(D)** Effect of CO on the endogenous fluorescence spectra of MP **(E)** Effect of CO on the ultraviolet absorption spectrum of MP **(F)** Effect of CO on the ultraviolet second-derivative spectrum of MP.

As shown in Figure 2A, (Note: m is Markers; 1 is MP, 2 is 0.15% CO-MP, 3 is 0.30% CO-MP, 4 is 0.45% CO-MP, and 5 is 0.60% CO-MP), troponin T (about 34 kDa), actin (about 45 kDa), myosin heavy chain (MHC, about 200 kDa), paramyosin (about 100 kDa), as well as tropomyosin (about 38 kDa) make up the majority of MP, which is corroborated by additional studies somewhat on typical MP protein spectrum (22). The intensity of all bands identified in MP decreased with increasing CO content, suggesting that these subunits are involved in Maillard reactions with CO and MP. After CO modification, the molecular weight of MHC decreases, which may be since insoluble aggregates or cross-linked compounds induced by CO cannot enter the stacked gel (9, 23). While actin, tropomyosin, and troponin T bands migrate upward, indicating that a large molecular weight product was formed. The mobility of electrophoretic bands only depends on the molecular weight of protein subunits. Similar discoveries demonstrated that the CO addition postponed MP’s aggregation (12, 17).

### 3.2. Spectral analysis

#### 3.2.1. Fourier transform infrared spectroscopy analysis

FTIR was incorporated to examine water-soluble MP’s, CO’s microstructures, and also their compounds, at the same time, we tried to discuss more and potential intermolecular interactions’ features (i.e., electrostatic interactions and hydrogen bonds). Because of stretching bands of -NH’s overlapping, a broad absorption peak of CO appeared near 3,435 cm^−1^ of the -OH group, and the absorption bands of the C-H bond at position 2,885 cm^−1^ manifested in a mild maximum. As shown in Figure 2B, amide I, II as well as III all have had the primary absorbing reaching a maximum of MP at 1655 cm^−1^ (amide I) for C=O stretching, 1,544 cm^−1^ for N–H bending, also 1,397 cm^−1^ for C–N stretching and N–H bending, respectively. O-H and N-H organizations, both released and attached, were capable of establishing hydrogen bonds including the carbonyl group of such peptide interconnection in proteins, which resulted in the broad band noticed in the 3,500–3,000 cm^−1^ zone. The 3,500–3,000 cm^−1^ range broad band was attributable to free and bound O–H and N–H groups, which were able to form hydrogen bonding with the carbonyl group of the peptide linkage in proteins (17). The amide II and III bands of the CO-MP complexes were shifted toward higher wavenumbers than MP, amide II moved from 1,544 to 1,586 cm^−1^, amide III moved from 1,397 to around 1,411 cm^−1^, indicating that the carboxylic acid group of MP (-COO) was closely related to CO. There are electrostatic interactions between (-NH_3_^+^)‘s amino units (31, 32). Additionally, MP demonstrated broadband at about 3,000–3,600 cm^−1^, and the broadband was more obvious with the increase of CO addition, indicating the enhancement of the hydrogen bonds. The results showed that hydrogen bonds and electrostatic interaction participated in MP with CO directly, also MP’s secondary structure has been altered through CO (33).

A recognized characteristic absorption peak of the secondary structure is the amide I. Protein’s secondary structure has mostly been upheld by noncovalent interactions forcing. When the environment changes, protein molecules rearranges to capture the smallest power and preserve a largely equilibrium point (32). It had been protein secondary structure’s recognized characteristic peak that is the amide I range (1,600 ~ 1700 cm^−1^), primarily closely linked towards C=O’s stretching vibration according to Figure 2C.

After CO modification, except 0.60% group α-helix content increased slightly, indicating that 0.60% CO effectively maintained the stability of MP structure, which inhibited the exposure of water-soluble proteins and hydrophobic reissued by interacting with protein molecules (34); Except for 0.15 and 0.60% CO-MP, in every group β-turn’s quantity increased; while β-sheet’s percentage in 0.15% also in 0.30% CO-MP was lower than that in the MP group. Similar results has shown in the study of Zhang et al. (35), the formation of the α-helix and β-turn structures is not beneficial for the formation of β-sheet nor structures which is random coil. Above changes tendency in secondary structure content indicated that the modification of chitosan oligosaccharide made the protein molecular structure more stable.

#### 3.2.2. Endogenous fluorescence spectrum analysis

As fluorescent compounds are generated, the Maillard process takes place as well, and these compounds could be the antecedents to brown colors (17). According to Figure 2D, native MP did not undergo fluorescence quenching, but after CO modification, with the increase of CO addition, the protein fluorescence intensity decreased significantly (*p* < 0.05), resulting in its fluorescence quenching, while its maximum the absorption peak (λ_max_) and peak shape remained unchanged. The fluorescence intensity varied as the Maillard reaction proceeded. After CO modification, the quenching constant (K_SV_) increased with the increase of CO addition, and the quenching of 0.60% CO-MP sample was the highest (Table 1). The fluorescence quenching process is generally divided into dynamic quenching and static quenching. At room temperature 25°C, when KS > 2.0 × 10^10^ l • mol^−1^ • s^−1^, static quenching happens, by contrast, it could turn out to be dynamic quenching (36). The contribution about CO on MP fluorescence quenching belongs to dynamic fluorescence quenching was illustrated by KS < 2.0 × 10^10^ l • mol^−1^ • s^−1^. Following above consequence, it could be concluded that the binding force between oligosaccharide molecules and MP surface groups was not strong, and stable ground-state complexes were not formed, which could only be formed through source of low bonding involving hydrogen bonds as well as hydrophobic interactions.

**Table 1:** Effect of chitosan oligosaccharide (CO) on the related parameters of myofibrillar protein (MP) fluorescence quenching.

Groups	Peak	KSV	KS
MP	585	-	-
0.15% CO-MP	505.76	128.39	1.04 × 10^10^
0.30% CO-MP	371.57	198.42	1.91 × 10^10^
0.45% CO-MP	341.2	200.25	1.59 × 10^10^
0.60% CO-MP	262.27	232.37	2.05 × 10^10^

#### 3.2.3. UV absorption spectrum analysis

Protein’s UV absorption spectrum is mostly caused due to the reason that tryptophan and tyrosine’s side chain units being exposed to UV light. The difference in the UV absorption spectra of the protein can be used to extrapolate the shape change of the protein. One or much aromatic amino acid residues may experience alterations in their surroundings as a result of conformational and dissociative changes as well as protein degradation (37). According to Figure 2E, the maximum UV absorption peak increased with the addition of CO, but the peak did not shift obviously, indicating that CO modification did not change the micro-environment around Tyr. The addition of CO led to the enhancement of the maximum UV absorption value, indicating that CO changed the molecular structure of MP, exposing more Trp and Tyr residues, and this trend grew stronger with the content of oligosaccharides (38).

With the increase of CO addition, the maximum absorption peak in the UV absorption spectrum increased, which proved that CO could make protein tertiary structure alter as a result. The resulting UV second derivative allowed for the identification of alterations in MP’s tertiary structure. This method is widely used for the conformational identification of protein molecules. As shown in Figure 2F, there were two positive absorption peaks near 289 nm and 296 nm (Trp and Tyr act together), and two peaks of negative absorption at 285 nm and 292 nm (Trp act). The value of r depends on the relative amount of Trp and Tyr and the amount exposed to the aqueous phase, i.e., *r* = *A* / *B* (39). The value of r for MP increased with increasing CO addition, while 0.6% CO-MP showed a decreasing trend, but numerical value was also significantly higher than MP (Table 2).

**Table 2:** Effect of chitosan oligosaccharide (CO) on the second derivative *r* value of myofibrillar protein (MP).

Groups	*r* value
MP	1.62 ± 0.09^a^
0.15% CO-MP	1.84 ± 0.05^ab^
0.30% CO-MP	1.91 ± 0.04^a^
0.45% CO-MP	1.98 ± 0.12^a^
0.60% CO-MP	1.96 ± 0.12^a^

It has been shown that: for Tyr, the value of “*r*” increases with increasing solvent polarity, while for Trp, the value of “*r*” is almost independent of solvent polarity; furthermore, the value of “*r*” and the positions of the peaks and troughs are functions of the relative amounts of Tyr and Trp and the average polarity of the two amino acids in the environment (40). “*R*” values for any sample were higher than those for native protein, which suggested that Tyr and Trp residues had been exposed toward a hydrophobic milieu, possibly as a result of the protein depolymerization (40). The higher r value indicated that during the depolymerization and unfolding of such molecular structure, MP’s latent tyrosine residues eventually appeared on the surface and move to the hydrophobic region and change their three-dimensional position (41).

### 3.3. Structural changes at the molecular scale

#### 3.3.1. Sulfhydryl content

**Figure 3:**
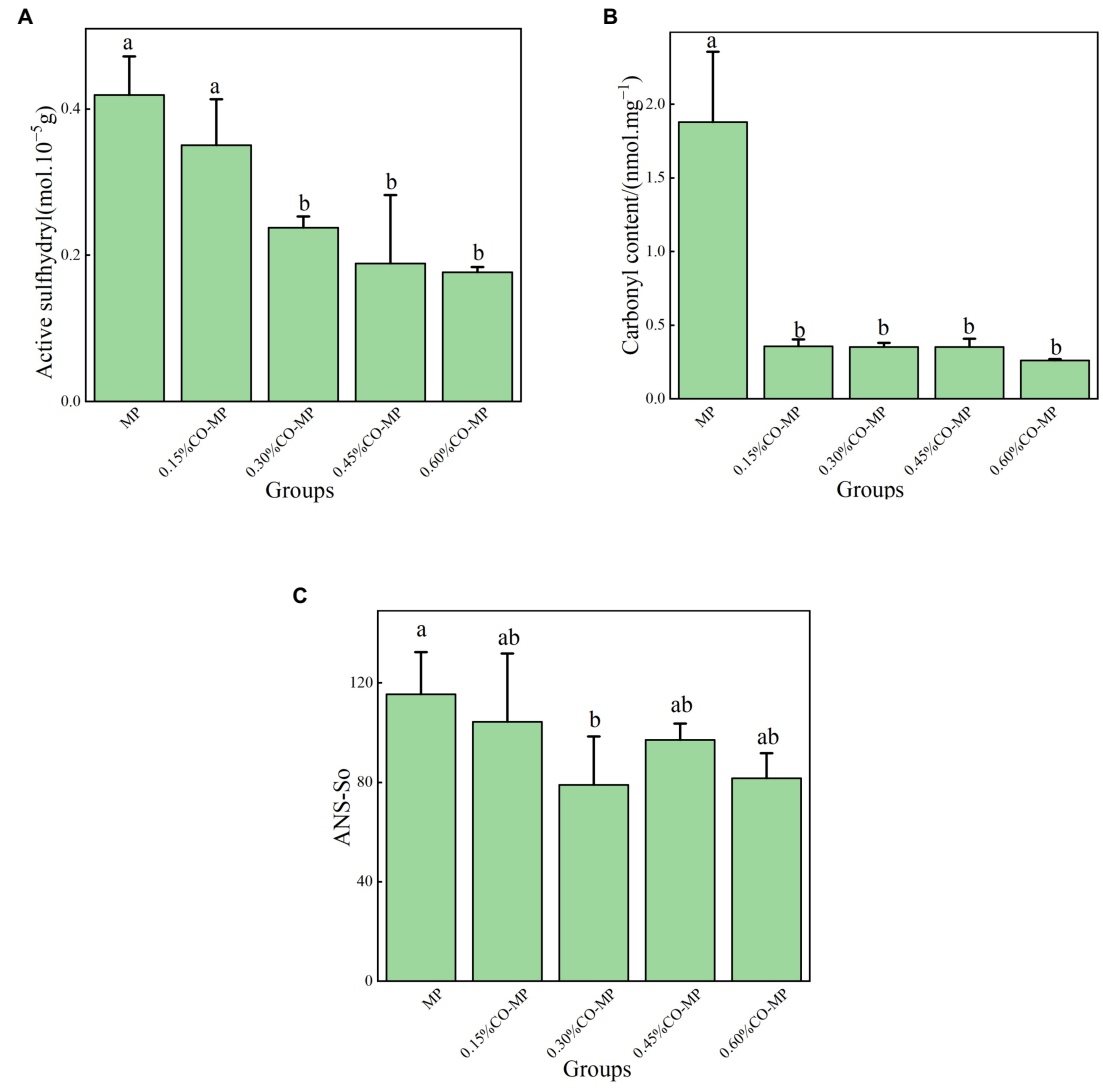
**(A)** Effect of chitosan oligosaccharide (CO) on the active sulfhydryl content of myofibrillar protein (MP) **(B)** Effect of CO on the carbonyl content of MP **(C)** Effect of CO on the surface hydrophobicity of MP.

The presence of sulfhydryl (SH) is an sign of how oxidized a protein is. The abundant SH units found in myofibrillar proteins are quickly converted to disulfide sites, resulting in reduced surface or total SH content. MPs are vulnerable to physical and chemical modifications and can be used to evaluate their exposure to protein surfaces (33). According to Figure 3A, the MP’s active SH group content dropped significantly with the increase of CO addition (*p* < 0.05). These results showed that after CO modification, the exposure of MP SH units was delayed, and MP’s oxidation was delayed to enhance protein’s identity (22). This matched with the carbonyl study results.

#### 3.3.2. Carbonyl content

Notably major changes to oxidized muscle protein is protein carbonylation, and oxidation’s rate is usually measured in the number of carbonyl compounds. MP goes through a variety of modifications during storage, involving conformational changes and accumulation, especially myofibrillar oxidation, which results in significant alterations, particularly the creation of carbonyl compounds (42). According to Figure 3B, after CO modification, the carbonyl quantity of myofibrillar protein was considerably reduced (*p* < 0.05), and 0.60% CO-MP’s carbonyl quantity was lower.

MP’s carbonyls, which were produced by certain amino acid side chains, were linked to structural modifications that lead to disintegration, accumulation, solubility, and functional loss. Hydrogen bonds occur between cationic amino acids’ polarity residues, oligosaccharides could take place in some of the water molecules that were present at the protein surface. This was in accordance with studies by Zhang et al. (22), which discovered that a particular pathway of interaction among oligosaccharide and shrimp MP. This supposed that in charge of the amount of oligosaccharide could control protein carbonyl during frozen storage (or processing), and oligosaccharide inactivated oxidation, acted as a scavenger of free radicals, and inhibited the formation of specific protein carbonyl as a metal chelating agent. It needed to be further verified and analyzed by experiments.

#### 3.3.3. Surface hydrophobicity

Analyzing hydrophobic residues’ dispersion which is disclosed over protein’s exterior, something that is strongly attributable to the usable characteristics, is an excellent approach for assessing the protein’s surface hydrophobicity (43). From Figure 3C, the surface hydrophobicity gradually decreased with the increase of CO addition, which indicated that the hydrophobic portion of the molecule was exposed and conformation changed. Protein gather might result from hydrophobic interactions among uncovered hydrophobic residues. Therefore, the addition of CO directly affected the conformational changes of protein molecules. It has been demonstrated that the addition of carrageenan oligosaccharides could postpone the rise in surface hydrophobicity and limit overall oxidation of MP, bind to functional groups in MP with the ionic or hydrogen bonds, forming a protective barrier and inhibiting the oxidative denaturation of proteins (22). Both oligosaccharides and MP were hydrophilical, overall findings from this investigation revealed that chitosan oligosaccharide may form protection of MP and retard oxidative protein denaturation.

### 3.4. Changes in functional characteristics

#### 3.4.1. Solubility and turbidity

**Figure 4:**
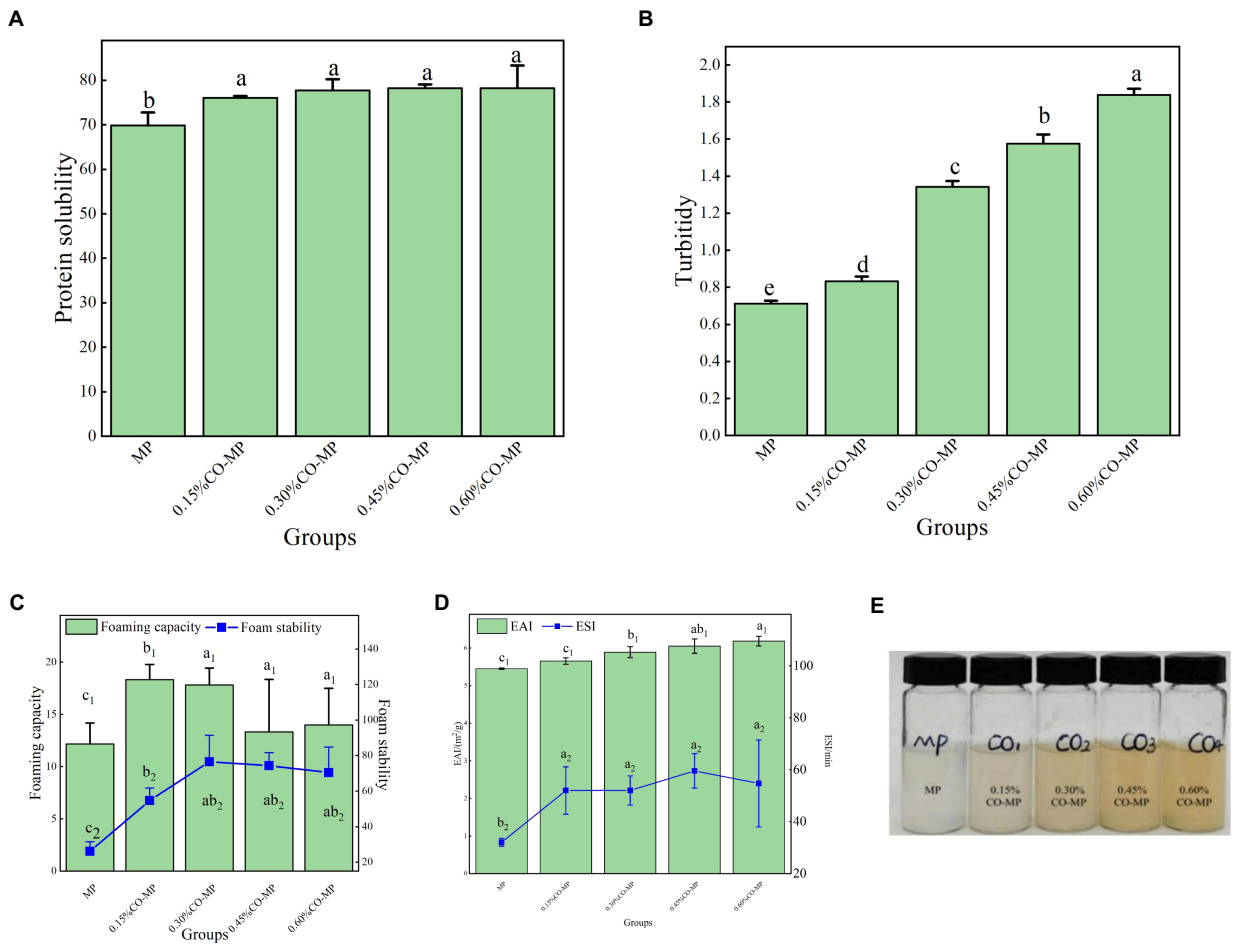
**(A)** The influence of chitosan oligosaccharide (CO) on the solubility of myofibrillar protein (MP) **(B)** The influence of CO on the turbidity of MP **(C)** The influence of CO on the influence of foaming capacity and foam stability of MP **(D,E)** The influence of CO on the emulsifying activity index (EAI) and emulsion stability index (ESI) of MP.

From Figure 4A, the MP’s solubility greatly rised following CO modification (*p* < 0.05), but the solubility of MP was not severely affected by the amount of CO. CO is attached to the surface of MP, which makes the system have more hydrophilic groups, which can enhance MP’s affinity with water molecules and inhibit the connection within MP molecules, boosting the solubility (43).

Alteration in hue is a prime determinant of how much the Maillard reaction happens (44). As shown in Figure 4B, after CO modification, the turbidity of myofibrillar protein increased considerably which is following adding more and more CO (*p* < 0.05). MP solution’s color was deepened, on the one hand, because CO itself was pale yellow, and on the other hand, some chemical changes occurred in CO and MP in the solution. This was in accordance with the finding that carbonyl and sulfhydryl groups were significantly reduced in MP and also suggested that a Maillard reaction might occur between MP and CO (17).

#### 3.4.2. Foaming capacity and foam stability

Foam is a colloidal framework of gas in a solution that has a tendency to suddenly split and is trapped by the composition layers that rises to the top due to density variations. Foam is an important indicator of emulsion performance (45). Figure 4C, the better foaming ability and stability were partly a result of the higher solubility brought on by the addition of CO (*p* < 0.05), and the performance was better at 0.15%–0.30%.

The foaming properties of emulsifiers are related to their spreading and absorption capacity at the air-water edge. Hence it could possibly be concluded which the saccharification of MP with CO encouraged the development of a film around the bubbles in the aqueous dispersion phase and inhibited melding (9, 46). What’s more, since protein’s solubility had a significant impact on the dielectric behavior, the increased solubility by the application of CO brought and contributed to the better foaming ability and stability.

#### 3.4.3. Emulsifying activity index and emulsion stability index

A protein’s capacity to lower the interfacial stress between two normally incompatible liquids is what gives its emulsifying properties. As shown in Figures 4D,E, the emulsification of MP climbed dramatically, well with application of more CO amount (*p* < 0.05), and 0.60% CO-MP showed higher emulsification ability. Most of the time, the reduction of the emulsifier interfacial tension was created by aiming the emulsifier’s hydrophobic and hydrophilic components at the polar element and non-polar part (oil phase) (water phase). Given the greater solubility that resulted from the addition of CO, CO-MP demonstrated a greater ability to emulsify in this investigation, which made it easier to use at the oil–water interface. What’s more, because of steric hindrance of CO, it was feasible to avert the emulsion’s spread phase droplets coalescing, thus maintaining the durability of the emulsion (9).

### 3.5. Particle size distribution

**Figure 5:**
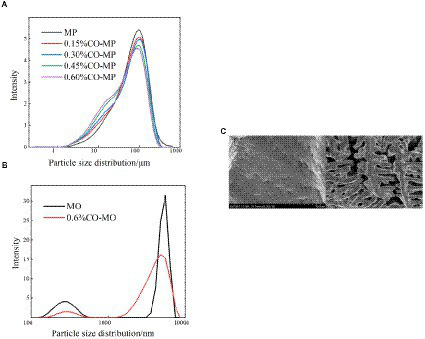
**(A)** The influence of chitosan oligosaccharide (CO) on the particle size of myofibrillar protein (MP) **(B)** The effect of 0.60% CO on the particle size of myosin (MP) **(C)** SEM of myosin.

The results of droplet diameter reveal that interface area is one of the important factors affecting particle size (26), and The interaction among proteins and small molecules within lotion has been confirmed by numerous investigations using droplet diameter (46). To learn more regarding the protein’s microstructure, using the laser particle size scanner. From Figure 5A, there was a particle size peak at 92.01 μm, MP was at 92.01 μm. After CO modification, with the increase of CO addition, the peak value at MP decreased, and the size was smaller gradually, which decreased significantly in every CO group. As shown in Table 3, after CO modification, the volume average diameter d_4,3_ of MP samples drastically dropped with the growth in CO, while 0.60% CO-MP samples were significantly different from 0.15% CO-MP and 0.30% CO-MP samples (*p* < 0.05), and there wasn’t a great deal of a distinction here from 0.45% CO-MP samples (*p* > 0.05).

**Table 3:** The effect of chitosan oligosaccharide (CO) on the average diameter d_4,3_ and d_3,2_ of myofibrillar protein (MP) droplets.

Groups	*d*_4,3_ (μm)	*d*_3,2_ (μm)
MP	92.01 ± 2.09^a^	37.13 ± 1.19^a^
0.15% CO-MP	80.72 ± 0.91^b^	30.38 ± 1.70^b^
0.30% CO-MP	77.03 ± 0.04^c^	28.37 ± 0.38^b^
0.45% CO-MP	73.98 ± 0.25^cd^	27.31 ± 1.82^b^
0.60% CO-MP	71.38 ± 0.97^d^	21.88 ± 3.59^c^

After CO modification, the average diameter d_3,2_ of MP area drastically shrank with an upsurge in CO supply (*p* < 0.05), and d_3,2_ of 0.60% CO-MP sample was massively reduced which is the totally different with the remaining (*p* < 0.05), but still no statistically noteworthy distinction was identified among 0.15% CO-MP, 0.30% CO-MP and 0.45% CO-MP (*p* > 0.05).

After CO modification, the interfacial activity of MP was significantly increased, the interfacial adsorption speed was accelerated, and the droplet size of the lotion was significantly reduced. MP could be more effectively and easily located on the oxygen/water interface (26). The results demonstrated that the mixture’s particle size gradually decreased with the increase of CO addition, inhibiting protein aggregation (12), and MP has higher stability (47). The reduction in particle size suggested that CO and MP particles had generated a soluble combination that resisted protein denaturation. This behavior could be due to the strengthening of electrostatic repulsion following CO alteration. Moreover, the presence of CO might make the mixture viscous, lower the collision frequency of particles and potentially hinder the development of large protein–protein clumps.

### 3.6. Structural changes at the microscopic scale

#### 3.6.1. AFM changes of myosin after CO modification

The study of dietary proteins has made excellent and widespread by utilizing atomic force microscopy. The most common atomic force microscope application approach for studying dietary proteins is nanoimaging. The morphological and structural characteristics of meat protein have been extensively studied to determine the composition, tenderness, and toughness of the tissue (48).

We observed morphological changes with AFM and evaluated the effect of CO on myosin in macromolecular crowding environment. The difference in mean roughness could be used to measure the amount of protein gather. Table 4 revealed that the layer of untreated myosin was rough, 0.60% CO reduced the surface roughness and height of myosin. The morphological structures of MO and 0.6% CO-MO mixtures were shown in Table 4. It could be seen from the 3D and 2D images that the surface of untreated MO had a rough surface; the morphology of MO aggregates was quite different from that of 0.6% CO-MO, which indicated that CO affected the surface morphology of MO, made the MO surface smoother and delaying the aggregation of MO. The results showed that myosin’s morphology was impacted by CO, which also smoothed out its exterior as well as slowed down myosin’s tendency to aggregate. This outcome might be the result of CO-MO being assembled into base pairs or multimers, leading to a consequence which shows poor unfolding of MOs, worse grouping of MOs, and inferior clustering heights of MOs (49).

**Table 4:** Atomic force microscope images of chitosan oligosaccharide (CO) and myosin (MO).

	2D	Height	3D	Roughness
CO	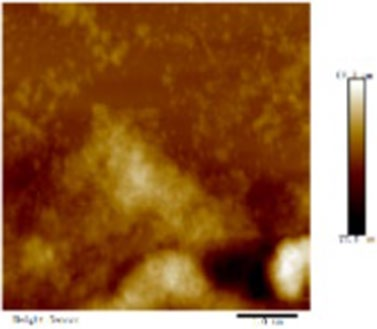	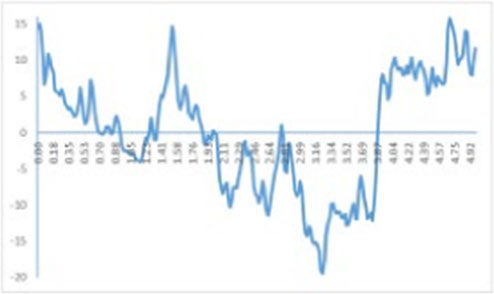	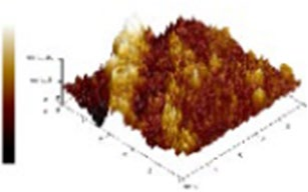	4.91 ± 0.77
0.60% CO-MO	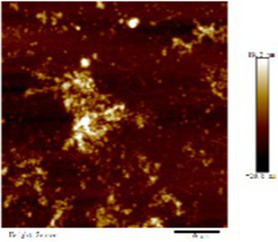	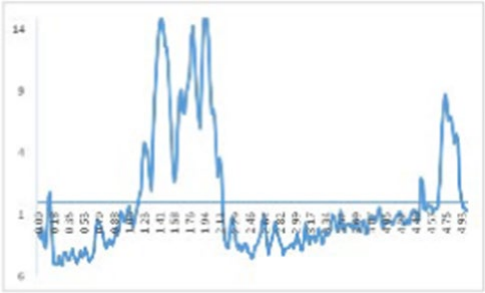	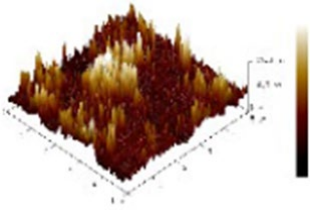	3.72 ± 0.40

#### 3.6.2. DLS changes of myosin after CO modification

MO sample demonstrated the same trend as the MP sample. The particle size distribution of 0.60% CO-MO complex moved to smaller particles, as shown in Figure 5B: MO sample showed double peaks in the figure. After CO modification, both peaks decreased. The peak at MO shifted, revealing that the CO-MO complex’s size was distributed to small particles. The DLS results showed the obvious unfolding of myosin, and the CO-MO complex had higher stability (50). This further proved and demonstrated that the particle size’s became smaller and inhibited protein aggregation because of a formation of CO-MO complex (12).

#### 3.6.3. SEM changes of myosin after CO modification

The SEM microstructure of CO-treated MO was compared with that of MO as shown in Figure 5C. The MO structure closely resembled a thin cloth with particles of different sizes on it, while in the CO-MO coacervate, vacuoles of various sizes were spaced apart in a permeable system structure. Changes could result from the MO losing its internal structure and suffering bodily harm from the ice crystals that are being produced. A sophisticated compressed system was formed through the collision of two macromolecules with antagonistic energies, trapping water droplets in the condensed phase and boosting the intricacy of such organization (33).

## 4. Discussion

The effect of chitosan oligosaccharide on myofibrillar protein denaturation of silver carp during cold storage was studied. Chitosan oligosaccharide’s addition could greatly decrease capacity average diameter of MP (d_4,3_) and space average diameter of MP (d_3,2_) with increment of CO concentration; chitosan oligosaccharide and myofibrillar protein formed CO-MP complex through electrostatic interaction, which had better stability. This denser structure could make the exterior of myosin smoother as well as reduce the roughness of myosin. The complex’s fluorescence intensity dropped as the CO concentration increasing, but its higher absorption apex (**λ**_max_) and peak shape essentially stayed unaltered. The addition of CO enhanced the accessibility of tryptophan residues, but did not affect the tryptophan’s milieu. The CO-MP complex had good antioxidant properties, the addition of chitosan oligosaccharide could reduce the content of carbonyl, sulfhydryl oxidation, 0.60% group seemed to have a better effect, and the disulfide covalent bond is formed. The complex’s interfacial hydrophobicity diminished as CO concentration increase. The solubility and turbidity increased significantly. CO-MP had better foaming and foam stability, but the emulsifying performance was not obvious. It was speculated that the reason for this was that chitosan oligosaccharide has a substantial molecular mass.

The binding force between oligosaccharide molecules and MP surface groups was not strong, and stable ground state complexes cannot be formed, which only feeble non-covalent bonds, including hydrogen bonds and hydrophobic interactions could create. The relationship between MP and CO involved both electrostatic interaction and hydrogen bonds. CO’s addition could change molecular structure of MP, expose more Trp and Tyr residues. The exposure of MP sulfhydryl also delayed and the oxidation of MP, thus the quality of protein was improved.

Through establishing hydrogen bonds with charged amino acids’ polarity residues, CO could replace some of the liquid molecules and coat the exterior of proteins. The attachment of CO to the surface of MP caused more hydrophilic groups in the complex, which could inhibit the association of MP molecules, enhance their propensity for molecules of water and solubility. In addition, the higher solubility brought by the addition of CO also helped to strengthen MP’s foaming capabilities and stability. Because protein’s solubility had a positive correlation with the interface capability, the emulsification of MP increased significantly with the increase of the amount of CO, and 0.60% CO-MP had a higher emulsifying ability. The particle size’s dropping further indicated the creation of a protein-aggregation-inhibiting solvent complicated of CO and MP particles. This behavior could be due to the augmentation of electrostatic interactions following CO modification.

Microscopic studies of CO and myosin further verified that complexation blocked proteins against accumulating. CO-MO condensates showed a porous network structure with different sizes of vacuolar intervals. As well as 0.60% group working with MO showed better performance, the formation of a complex of CO and MO particles that inhibited protein aggregation, the CO-MO coacervate demonstrated a permeable network structure with heterogeneously size vacuoles intermingled, CO could improve and reduce the roughness of myosin.

## 5. Conclusion

Myofibrillar protein molecules’ rearrangements were directly influenced by CO modification, which also altered the ancillary structure of the protein, delayed MP polymerization, inhibited protein oxidation, stabilized the molecular structure of the protein, and enhanced its quality and functional capabilities. In particular, it increased solubility, improved foaming performance, foam stability and emulsification ability. The microscopic studies of CO and myosin further illustrated the above conclusions. Overall, chitosan oligosaccharide, as a naturally active macromolecule, changed the structure and function of proteins and raised the caliber of protein-based products. Especially for those kinds of fish products which are mainly applying the functional characteristics of myofibrillar protein, such as fried fish fillets, minced fish products, fish sausages and so on. In the future, due to different sugar donors could cause diverse improvement effects of proteins, researchers could do more research about the effect of various molecular weights of the sugar donors which will promote the development of relevant special performance products in the food processing field.

Moreover, we noticed that CO had a strong antioxidant capacity, which meant we might create a kind of materials that both own the anti-inflammatory characteristic and the fish protein’s nutritional value. By unexpectedly finding differences in mixing methods, as well reviewing the former studies, we learned that a way named wet-heating glycosylation showed better results, therefore we suggest future researchers could spend more effort on trying this measure and also optimize the experimental conditions to improve efficiency. Currently, 3D printing technology draws lots of attention from researchers, so we may use this emerging way to create more valuable and deeper exploration in this field. Thus, we could apply this technology to industrial production, which could significantly reduce energy consumption and production costs. Finally, it is still unclear for the mechanism of glycosylation which needs to be explored more in future studies.

## Data availability statement

The original contributions presented in the study are included in the article/Supplementary files, further inquiries can be directed to the corresponding authors.

## Author contributions

HC completed the writing part, scientific analysis and modification after the delivery of the manuscript, text modification, text and language editing after the review, etc. QW completed some data analysis and literature search. ZZ and HC draft the figures in the manuscript. HC, QW, ZZ, and JK revised the manuscript. HC was involved in the supervision and review of the entire experiment and thesis writing. All authors contributed to the article and approved the submitted version.

## Funding

This work was supported by the Key Research and Development Project of Hainan Province (ZDYF2022XDNY335); Opening Project of Key Laboratory of Coarse Cereal Processing of Ministry of Agriculture and Rural Affairs, Chengdu University (No. 2022CC10); Cooperation project of Amway China Co Limited and National University of Singapore (Suzhou) Research Institute (No. Am20220229RD); Class B funding of Shanghai Municipal Savings Plan (2022); Research Foundation for the Doctoral Promotion Program, Suzhou Polytechnic Institute of Agriculture (BS[2022]18); Special Project of High-tech Industrialization of Science and Technology Cooperation between Jilin Province and Chinese Academy of Sciences, Extraction, Purification and Industrialization of Type I Collagen of Medical Bovine Achilles Tendon.

## Conflict of interest

QW and JK were employed by Nutrilite Health Institute.

The remaining authors declare that the research was conducted in the absence of any commercial or financial relationships that could be construed as a potential conflict of interest.

## Publisher’s note

All claims expressed in this article are solely those of the authors and do not necessarily represent those of their affiliated organizations, or those of the publisher, the editors and the reviewers. Any product that may be evaluated in this article, or claim that may be made by its manufacturer, is not guaranteed or endorsed by the publisher.
